# Prevalence and factors associated with utilization of rehabilitation services among people with physical disabilities in Kampala, Uganda. A descriptive cross sectional study

**DOI:** 10.1186/s12889-019-8076-3

**Published:** 2019-12-27

**Authors:** Swaibu Zziwa, Harriet Babikako, Doris Kwesiga, Olive Kobusingye, Jacob A. Bentley, Frederick Oporia, Rebecca Nuwematsiko, Abdulgafoor Bachani, Lynn M. Atuyambe, Nino Paichadze

**Affiliations:** 1 Department of Disease Control and Environmental Health, Makerere University School of Public Health, College of Health Sciences, New Mulago Hill Hospital Complex, P.O Box 7072, Kampala, Uganda; 2 Department of Maternal and Child health, Makerere University College of Health Sciences, Kampala, Uganda; 3 Department of Health Policy, Planning and Management, Makerere University School of Public Health, Kampala, Uganda; 4 Department of International Health and International Injury Research Unit, Johns Hopkins Bloomberg School of Public Health, Baltimore, MD USA; 5 Department of Global Health, Milken Institute School of Public Health, the George Washington University, Washington, DC USA

**Keywords:** People with physical disability, Rehabilitation services and Kawempe division

## Abstract

**Background:**

Worldwide, fifteen percent (15%) of the world’s population or one (1) billion people live with some form of disability. In Uganda, 12.4% of the Uganda’s population lives with some form of disability and Kawempe division accounts for (22.6%) of all persons with disabilities living in Kampala district. Rehabilitation services are provided within Kawempe division at Mulago hospital physiotherapy department and Katalemwa rehabilitation center in Kampala district, Uganda at a free and a subsidized cost to help to improve the function, independence, and quality of life of persons with physical disabilities. However, many people with physical disabilities do not utilize the services and the reasons are not clear.

**Methods:**

The study design was a descriptive cross-sectional study employing quantitative methods of data collection. A total of 318 participants were included in the study. Simple random sampling was used to select the study participants. Ethical issues were maintained at all levels during data collection and dissemination of results.

**Results:**

The study revealed a prevalence of 26.4% of the utilization rehabilitation services among people with physical disabilities in Kawempe division, Kampala, Uganda. Factors that were significantly associated with utilization of rehabilitation services among people with physical disabilities at multivariable logistic regression analysis included; age (AOR: 0.30; 95% CI: 0.12–0.74), socioeconomic status (AOR: 2.13; 95% CI: 1.03–4.41), education level (AOR: 4.3; 95% CI: 1.34–13.91) and awareness of the participants about the rehabilitation services (AOR: 5.1; 95% CI: 2.74–9.54) at *p* value ≤0.05.

**Conclusion:**

The study revealed a prevalence of 26.4% of the utilization rehabilitation services among people with physical disabilities in Kawempe division, Uganda. Factors that were significantly associated with utilization of rehabilitation services included; age, socioeconomic status, education level and awareness of the participants about the services. Therefore, the government and other relevant stake holders should increase sensitization and awareness of rehabilitation services, their benefits and facilities providing such services to people with physical disabilities, healthcare professionals and the general public.

## Background

International Classification of Functioning and disability (ICF) defines disability as a permanent impairment, activity limitation and restriction in participation in any life situation resulting from any health condition whether congenital or acquired [1, 2]. A physical disability was defined as a permanent mobility, visual, speech and hearing impairment from any health condition whether congenital or acquired [1]. Rehabilitation is a set of interventions designed to optimize functioning and reduce disability in individuals with health conditions in interaction with their environment [3, 4]. A Health condition refers to disease (acute or chronic), disorder, injury or trauma [3, 5]. According to World Health Organization (WHO), rehabilitation is one of the essential components of the quality health services that should be included in Universal Health Coverage. This means that all individuals should be able to access quality rehabilitation services without fear of financial hardships [6, 7].

Worldwide, disability is a major public health concern. Globally, it is estimated that one billion people of the world’s population (15%) live with some form of disability [8].. About 110 to 190 million adults encounter substantial difficulties in their daily lives [8, 9]. In order to reduce the number of person with disabilities (PWDs), the WHO Member States in 2014 endorsed global disability action plan 2014–2021 with the aim to remove barriers and improve access to health services; strengthen and extend rehabilitation and community-based rehabilitation [8]. The plan was adopted in response to 66th World Health Assembly resolution on disability which urges member states to implement the recommendations of the United Nation’s Convention of the Rights of PWDs (CRPD); work towards the inclusion of all PWDs in health care; promote rehabilitation services across the life course and for a range of different health conditions with the goal of attaining good health for all PWDs [10, 11].

Furthermore, the WHO Member States, international and professional organizations, non-governmental organizations and rehabilitation experts on 7th February 2017 issued Rehabilitation 2030; call for action [3, 12]. The major aim for bringing together these stakeholders was to inspire them to actively participate in a global effort toward strengthening rehabilitation in health systems, improving rehabilitation governance and investment; expanding a high-quality rehabilitation workforce; and enhancing rehabilitation data collection [3, 6]. WHO’s call for action not only underscores the increasing need for rehabilitation, but also brings awareness to the role rehabilitation can play in achieving UN’s Sustainable Development Goals (SDGs), specifically in ensuring that all people all over the world are able to experience good health and well-being [3, 13].

The Global Burden of Diseases (GBD), Injuries, and Risk Factors Study 2017 includes a comprehensive assessment of incidence, prevalence, and years lived with disability (YLDs) for 354 causes in 195 countries and territories from 1990 to 2017 [14, 15]. Previous GBD studies have shown how the decline of mortality rates from 1990 to 2016 has led to an increase in life expectancy, an ageing global population, and an expansion of the non-fatal burden of disease [14, 16]. Rehabilitation optimizes functioning and supports those with health conditions to remain as independent as possible, to participate in education, to be economically productive, and fulfill meaningful life roles [3, 4].

The burden of disability is unevenly distributed between low and high income countries with low and middle income countries carrying the greatest burden of disability compared to high income countries. Globally, it is estimated that there over 600 million PWDs of whom 400 million live in developing countries and about 80 million live in Africa [5, 8, 17]. United Nation’s report maintains that about 40% of Africa’s population consists of PWDs, including 10–15% of school-age children [18] . This percentage would translate into about 300 million people with PWDs in Africa [18]. In Africa, data on utilization of rehabilitation services is still very limited. A population based study conducted among 33 countries in LMIC revealed generally limited access to rehabilitation services. In many countries, rehabilitation centers had collapsed or ceased function and others were not operating adequately [19]. In addition, an extensive survey of rehabilitation doctors in Sub-Saharan Africa identified only six, all in South Africa, for more than 780 million people, while Europe has more than 10,000 and the United States has more than 7000 [20]. Furthermore, a survey of national societies of physical and rehabilitation medicine in Africa revealed that apart from the Northern African countries of Morocco, Tunisia, and Algeria which started the practice of rehabilitation in the twentieth century, almost all of the very few Sub-Sahara African countries with physical and rehabilitation medicine associations were formed in the early twenty-first century. In addition, to the countries with National Societies, there are some countries such as Benin, Ghana, Mali, Ethiopia, South Africa, and Uganda which do not have National Societies [21].

People with disabilities encounter several challenges in accessing rehabilitation services such as poor accessibility, high cost of accessing the services including transport costs, lack of awareness of the need for rehabilitation services, ignorance and long waiting times [6, 7]. In addition, other factors include; lack of well-established national policy on physical rehabilitation, inadequate service provision, poor infrastructure development, lack of enough trained physical therapist professionals, misconception and traditional beliefs [6, 22, 23]. People with disabilities encounter numerous challenges in the societies including lack of accessible health facilities, poor education, unemployment, and high transport costs. People with disabilities are also known to have poor health, poor socioeconomic status, lower education levels which limit their participation in economic activities in the society than abled people [10, 24].

Many of the challenges encountered by PWDs in the societies including discrimination in service delivery, stigmatization from the wider community, discrimination in employment and business can be avoided [5, 8]. Improved access to health care integrated with rehabilitation services generally improves health and well-being of PWDs including attaining better education, better employment, caring and participating in family, community and public life [6]. Increased access to rehabilitation services also lead to better overall socio-economic outcomes for people with disabilities and achievement of broader global development goals [10, 25].

The disease burden in Uganda is steadily increasing with communicable diseases including; Malaria, HIV/AIDS, TB, respiratory tract infections, diarrhoea, and immunizable diseases accounting to over 50% of the total morbidity and mortality [14]. Uganda like other developing countries is also experiencing an epidemiological transition characterized by an increasing burden of non-communicable diseases (NCDs) including cancer, diabetes, cardiovascular diseases, violence and injuries especially road traffic injuries accompanied by population ageing [14, 15]. The increasing double burden of diseases both communicable and non-communicable diseases in Uganda has resulted to the increase in the number of persons with disabilities [26, 27]. Rehabilitation is relevant for persons experiencing or likely to experience limitations in everyday functioning due to aging or health conditions, including chronic diseases or disorders, injuries or trauma [28].

According to Uganda Population Based Survey, the overall disability prevalence rate for population aged 2 years above stands at 12.4%. Sex difference reveals that disability is higher among women (14.5%) compared to men (10.0%) and the disability prevalence rate was higher among those living in urban areas (15.0%) compared to those in the rural areas (12.0%) [29]. People with disabilities are one of the vulnerable groups of the population in Uganda and continue to face numerous challenges in terms of abuse and violations of their human rights [30, 31]. Among the major types of rights violations are sexual abuse, discrimination in service delivery, stigmatization from the wider community, discrimination in employment and business and generally marginalization in terms of the economic, social and political rights [30, 31]. However, data on utilization of rehabilitation services to guide comprehensive health policy formulation at national and regional levels is still scarce.

Kawempe division is located in the Northern part of Kampala capital city (KCC) of Uganda. Due to the fact that the division is found in a low lying area of Kampala city with many informal settlements, residents in the slum areas of the Division must cope with natural location hazards like floods, garbage dumps, busy roads, power lines, open drains, water bodies, sinking soil industrial hazards, evictions, crime and community violence which increases their vulnerability to injuries and disabilities [32]. A report from Uganda Bureau of Statistics shows that Kawempe division accounts for 22.6% of all PWDs living in Kampala district [29]. Rehabilitation services are provided within Kawempe division at Mulago hospital physiotherapy department and Katalemwa rehabilitation center at a free and a subsidized cost to help improve the function, independence, and quality of life of people with physical disabilities. However, many people with physical disabilities do not utilize the services and the reasons are not known. There is also limited information about the prevalence of utilization of rehabilitation services among people with physical disabilities. Records from Mulago physiotherapy department and Katalemwa rehabilitation center shows that only 15.2% (3000 out of 19,776) PWDs in the division had utilized the services. However, results from facility records cannot be representative of PWDs utilizing rehabilitation services in the division because Mulago physiotherapy department and Katalemwa rehabilitation center serves a wide geographical area with Mulago hospital acting as national referral hospital of Uganda. In addition, such data is continuously being collected without analysis done over years to estimate the actual prevalence of utilization of rehabilitation services. Furthermore, the facility records captures all types of disabilities not only limited to physical disabilities which further makes it difficult to estimate the actual prevalence of utilization of rehabilitation services among people with physical disabilities. Therefore, the present study was conducted to determine the prevalence and factors associated with utilization of rehabilitation services among people with physical disabilities in Kawempe division, Kampala Uganda.

## Methods

The study was conducted in Kawempe division. The division is located in the Northern part of Kampala the capital city of Uganda. The division is one of the five Divisions that make up Kampala City. Administratively, the division is made up of fifteen parishes [32]. Rehabilitation services are provided within Kawempe division at Mulago hospital physiotherapy department and Katalemwa rehabilitation center. Both facilities provide a wide range of services for mobility, vision, speech or communication and hearing impairments which include;- Therapeutic exercises modalities**;** Exercise table, Training stairs (steps), Weights (dumb bell, sand), Exercise bikes (stationary exercise bikes), Exercise (active and passive), Massage, Heat, Ice and Joint Mobilization. Assistive device technologies; Crutches, Wheel chairs, Walkers, Clappers, Prosthetics (artificial legs or arms), Orthotics (clappers, splint, brace, shoe insert or cast) for mobility impairment. Hearing aids, Cochlear implants for hearing impairment. Magnifiers, Ocular devices, Talking books (talking dictionaries), Large print and talking calculators, Closed circuit televisions (CCTV), Software such as screen reading and text enlargement programs for vision impairment Communication boards and Speech synthesizers for Speech or Communication impairments. Multidisciplinary rehabilitation service providers for both facilities include; − occupational therapists, orthotists and prosthetists, physiotherapists, psychologists, social workers, speech and language therapists.

The study population comprised of 318 people with physical disabilities aged 2 years and above living in Kawempe division. Physical disabilities included persons with permanent mobility, visual, speech and hearing impairment from any health condition whether congenital or acquired. The study design was a descriptive cross-sectional study employing quantitative method of data collection. The sample size for this study was calculated using Kish Leslie (1964) formula for a single proportion. The study employed simple random sampling technique to select study respondents using computer generated random numbers. The people with physical disability were identified using Washington Group on disability statistics which has six questions related to six functional domains which include; mobility, hearing, visual, speech, self-care and cognition [33]. The tool was used in Uganda Demographic Health Surveys to identify PWDs [29, 34]. The sampling frame with 1270 people with physical disabilities was obtained from Kawempe Division Disabled Community (KDDC) office located in Kamwokya along Mawanda Road which is working under the National Union of Disabled Persons of Uganda (NUDIPU). In order to reach out to the study participants, the leaders of PWDs disabled union helped to trace the study respondents in the division.

The dependent variable of the present study was prevalence of utilization of rehabilitation services which was defined as use of any rehabilitation service at any point in life. The overall prevalence of utilization of rehabilitation services was recorded as a binary variable where those who used rehabilitation services at any point in life responded “Yes” whereas those one who never used rehabilitation services responded “No”. The same approach was used in a similar study conducted in Brazil [35]. The independent variables included; personal factors (age, sex, comorbidity conditions, education, income or socio economic status, motivation, attitudes and degree of disability) and environmental factors (accessible physical environment, accessible services, availability of caring family, attitude of health professionals, accessible transport, cost of services, waiting hours, availability of services, distance to the facility, accessible information, disabled people’s organization and social attitude). Personal and environmental factors were reported by International Classification of Functioning, Disability and Health (ICF) model as either facilitators or barriers to utilizing rehabilitation services [1].

Quantitative data was collected using interviewer administered questionnaire by trained research assistants. The English questionnaire was translated into the local language (Luganda) and then back translated to English to ensure that the translated version does not alter the meaning of the questions prior to their use. In order to measure socio-economic status, principal components analysis (PCA) was run on the 11 household assets evaluated. These assets included owning: (1) a radio, (2) a television, (3) mobile phone, (4) a bicycle, (5) a motorcycle, (6) a motor vehicle, (7) a piece of land (8) a manufactured bed (9) Plastered wall, (10) cemented floor (11) electricity. The sensitivity and reliability test was conducted on the 11 house hold items. Cronbach’s alpha coefficient was 0.8917 which was an implication that the variables were reliable to predict SES. The principal component on which most assets loaded was used to generate socio-economic status score for each participant. Participants were then grouped into SES quintiles (two descending groups). The same approach was used in Uganda Demographic Health Surveys [27, 34]. Education categories refer to the highest level of education attended, whether or not that level was completed. Education level of the study respondents was recorded as no education (not attended school or attained any level of education), primary, secondary and tertiary.

In order to measure distance; participants were asked the name of the nearby health facility with rehabilitation services. The exact distance of the respondents to the nearby facility with rehabilitation services was measured using Google earth. A distance of 5 km to the facility was recorded as an ideal distance and a distance above 5 km were recorded as a long distance to the facility. The same approach was used in Uganda demographic health surveys [27, 34]. Awareness of the study respondents about rehabilitation services was recorded as a binary variable by asking participants whether they have ever heard about physical rehabilitation services and facilities giving such services. Those who were aware of rehabilitation services answered “YES” and those who were not aware about rehabilitation services answered “NO”.

Data was checked and cleaned for mistakes before entered into Micro soft excel. Thereafter, data was exported to STATA version 13 for analysis. Data was presented using appropriate frequency tables using percentages or proportions. At Bivariate analysis, cross tabulation between the dependent variable (utilization of rehabilitation services) and the independent variables (potential factors associated with utilization of rehabilitation services) was performed at a time to determine their level of significance using a chi square and a *P* value < 0.05 at 95% confidence interval. In order to select the candidate variables to include in the model, bivariate analysis was conducted using logistic regression analysis with a *p*-values ≤0.1. Factors that were significantly associated with utilization of rehabilitation services at bivariate analysis and those that were not significant but with evidence from literature review indicating possible association with utilization of rehabilitation services was considered in the logistic regression model. Their respective Crude Odds Ratios (COR) were recorded at 95% confidence intervals. Then after; multivariate analysis was performed using the logistic regression model to determine factors that were significantly associated with utilization of rehabilitation services using backward elimination at *p* < 0.05. Their respective Adjusted Odds Ratios (AOR) were recorded at 95% confidence intervals. At this level, unknown and known confounding variables were controlled.

## Results

### Socio-demographic characteristics of people with physical disabilities in Kawempe division, Kampala, Uganda

A total of 318 study participants were included in the study. The mean age of the study participants was 27 years (SD ± 12.9) and the mean distance of the study participants to the nearest health facility with rehabilitation services was 9.8kms (SD ± 2.9). Nearly three quarter (69.5%) of the study participants were male. Less than half (40.5%) of the study participants were in the age range of 19 to 34 years. More than a half (52.52%) had attained primary education. Majority, (83.0%) of the study respondents were from low socioeconomic quintile and more than half (59.1%) of study participants had mobility impairments (Table 1).

**Table 1:** Socio-demographic characteristics of people with physical disabilities in Kawempe division

Variable	Frequency *n* = 318	Percentage (%)
Sex		
Male	221	69.5
Female	97	30.5
Age mean = 27(SD ± 12.9)		
2–18	99	31.1
19–34	129	40.6
35 above	90	28.3
Religion		
Catholic	47	14.8
Protestant	74	23.3
Christian Pentecostal	116	36.5
Muslim	81	25.5
Marital status		
Single	194	61.0
Married	46	14.5
Divorced/ separated	78	24.5
Education level		
None	44	13.8
Primary	167	52.5
Secondary	107	33.7
Socioeconomic quintiles		
Low	264	83.0
High	54	17.0
Occupation		
Not employed	144	45.3
Employed	174	54.7
Type of Disability		
Vision speech	72	22.6
Hearing	58	18.2
Mobility	188	59.1
Awareness		
No	200	62.8
Yes	118	37.1
Nature of disability		
Congenital	56	17.6
Acquired	262	82.4
Cause of disability		
Road traffic crashes	101	31.8
Infection	158	49.7
Other causes	59	18.6
Distance mean 9.8 km (SD ± 2.9)		
0-5 km	26	8.2
6 km above	292	92.0
Family support		
No	111	34.9
Yes	207	65.1

### Prevalence of the utilization of rehabilitation services among people with physical disabilities in Kawempe division, Uganda

The study revealed prevalence 26.4% of utilization of rehabilitation services. The prevalence of utilization of rehabilitation services was significantly different among study participants with different education levels; *p* = 0.001. In addition, the prevalence of utilization of rehabilitation services was significantly different among respondents with different socioeconomic quintiles and level of awareness about rehabilitation services respectively; (Table 2).

**Table 2:** Prevalence of the utilization of rehabilitation services among people with physical disabilities in Kawempe division

Variables	Utilization of physical rehabilitation services		*P* value
	Yes (%)	No (%)	
N = 318	84(26.4)	234 (73.6)	
Sex			
Female	30 (35.7)	67 (28.6)	0.227
Male	54 (64.3)	167 (71.4)	
Age			
35 above	16 (19.1)	74 (31.6)	0.003
19–34	47 (55.9)	82 (35.0)	
2–18	21 (25.0)	78 (33.3)	
Religion			
Muslim	21 (25.0)	60 (25.6)	0.289
Pentecostal	31 (36.9)	85 (36.3)	
Protestant	15 (17.8)	59 (25.2)	
Catholic	17 (20.2)	30 (12.8)	
Education level			
Secondary	51 (60.7)	56 (23.9)	0.001
Primary	27 (32.1)	140 (59.8)	
None	6 (7.1)	38 (16.2)	
Marital status			
Divorced/Separated	18 (21.4)	60 (25.6)	0.656
Married	14 (16.7)	32 (13.7)	
Single/Never married	52 (51.9)	142 (60.7)	
Occupation			
Employed	58 (69.1)	116 (49.6)	0.002
Not employed	26 (30.9)	118 (50.4)	
SES Quintiles			
High	28 (33.3)	26 (11.1)	0.001
Low	56 (66.7)	208 (88.9)	
Distance			
6 km above	74 (88.1)	218 (93.16)	0.146
0-5 km	10 (11.9)	16 (6.4)	
Disability			
Mobility	73 (86.9)	115 (49.1)	0.001
Hearing	5 (5.9)	53 (22.7)	
Vision speech	6 (7.1)	66 (28.2)	
Awareness			
Yes	59 (70.2)	59 (25.5)	0.001
No	25 (29.8)	175 (75.8)	
Nature			
Acquired	77 (91.7)	185 (79.1)	0.009
Congenital	7 (8.3)	49 (20.9)	
Cause of disability			
Others	11 (13.1)	48 (20.5)	0.001
Infection	20 (23.8)	138 (59.0)	
Road traffic crash	53 (63.1)	48 (20.5)	
Family support			
Yes	62 (73.8)	145 (62.0)	0.051
No	22 (26.2)	89 (38.0)	

Pearson’s chi square test(x^2)^, *P* < 0.05 are statistically significant

### Factors associated with utilization of rehabilitation services among people with physical disabilities in Kawempe division

At bivariate logistic regression analysis, factors that were significantly associated with utilization of rehabilitation services among people with physical disabilities included age, being in the age range of 19–34 years (COR: 2.13; 95% CI: 1.17–3.88) compared to being in the age range of 2–18 years, education level, having attained secondary school education (COR: 5.77 95% CI: 2.25–14.78) compared to no education, occupation, being employed (COR: 2.27; 95% CI: 1.34–3.85) compared to no employment, socio economic status, having a high social economic status (COR: 4.0; 95% CI: 2.17–7.36) compared to low socio economic status, type of disability, having a mobility impairment (COR: 6.98; 95% CI: (2.88–16.9) compared to vision or speech impairment, awareness, being aware of rehabilitation services (COR: 7.0; 95% CI: 4.03–12.17) compared to being un aware of the services, nature of disability, having acquired disability (COR: 2.91; 95% CI:1.26–6.72) compared to congenital disability. Cause of disability, having acquired a disability by an infection had 87% less odds of utilizing rehabilitation services (COR: 0.13; 95% CI: 0.71–0.24) compared to a road traffic crash. Other factors like sex, religion, marital status, and distance to the facility with rehabilitation services were not significantly associated with utilization of rehabilitation services (Table 3).

**Table 3:** Bivariate analysis of the factors associated with utilization of rehabilitation services among people with physical disabilities in Kawempe division

Variables	Utilization of physical rehabilitation services		Unadjusted OR (95%CI)	*P* value
	Yes (%)	No (%)		
Sex				
Female	30 (35.7)	67 (28.6)	1.38 (0.82–2.35)	0.228
Male	54 (64.3)	167 (71.4)	1.0	
Age				
35 above	16 (19.1)	74 (31.6)	0.8 (0.39–1.66)	0.553
19–34	47 (55.9)	82 (35.0)	2.13 (1.17–3.88)*	0.014
2–18	21 (25.0)	78 (33.3)	1.0	
Religion				
Muslim	21 (25.0)	60 (25.6)	0.62 (0.28–1.34)	0.223
Pentecostal	31 (36.9)	85 (36.3)	0.64 (0.31–1.32)	0.232
Protestant	15 (17.8)	59 (25.2)	0.45 (0.19–1.02)	0.056
Catholic	17 (20.2)	30 (12.8)	1.0	
Education level				
Secondary	51 (60.7)	56 (23.9)	5.77 (2.25–14.78)**	0.001
Primary	27 (32.1)	140 (59.8)	1.22 (0.47–3.17)	0.681
None	6 (7.1)	38 (16.2)	1.0	
Marital status				
Divorced/Separated	18 (21.4)	60 (25.6)	0.82 (0.44–1.52)	0.525
Married	14 (16.7)	32 (13.7)	1.19 (0.59–2.42)	0.620
Single/Never married	52 (61.9)	142 (60.7)	1.0	
Occupation				
Employed	58 (69.1)	116 (49.6)	2.27 (1.34–3.85)*	0.002
Not employed	26 (30.9)	118 (50.4)	1.0	
SES Quintiles				
High	28 (33.3)	26 (11.1)	4.0 (2.17–7.36)**	0.001
Low	56 (66.7)	208 (88.9)	1.0	
Distance				
6 km above	74 (88.1)	218 (93.16)	0.54 (0.24–1.25)	0.151
0-5 km	10 (11.9)	16 (6.4)	1.0	
Disability				
Mobility	73 (86.9)	115 (49.1)	6.98 (2.88–16.9)**	0.001
Hearing	5 (5.9)	53 (22.7)	1.0 (0.30–3.59)	0.953
Vision speech	6 (7.1)	66 (28.2)	1.0	
Awareness				
Yes	59 (70.2)	59 (25.5)	7.0 (4.03–12.17)**	0.001
No	25 (29.8)	175 (75.8)	1.0	
Nature				
Acquired	77 (91.7)	185 (79.1)	2.91(1.26–6.72)*	0.012
Congenital	7 (8.3)	49 (20.9)	1.0	
Cause of disability				
Others	11 (13.1)	48 (20.5)	0.2 (0.10–0.45)**	0.001
Infection	20 (23.8)	138 (59.0)	0.13 (0.71–0.24)**	0.001
Road traffic crash	53 (63.1)	48 (20.5)	1.0	
Family support				
Yes	62 (73.8)	145 (62.0)	1.72 (0.99–3.0)	
No	22 (26.2)	89 (38.0)	1.0	

*Bivariate logistic regression analysis, 95% Confidence Interval, *P* value< 0.05 are statistically significant

**Table 4:** Multivariate analysis of the factors associated with utilization of rehabilitation services among people with physical disabilities in Kawempe division.

Variables	Utilization of physical rehabilitation services		Unadjusted OR(95%CI)	P value	Adjusted OR(95%CI)	P value
	Yes (%)	No (%)				
Sex						
Female	30(35.7)	67(28.6)	1.38(0.82–2.35)	0.228		
Male	54(64.3)	167(71.4)	1.0			
Age						
35 above	16(19.1)	74(31.6)	0.8(0.39–1.66)	0.553	0.30(0.120.74*	0.009
19–34	47(55.9)	82(35.0)	2.13(1.17–3.88)*	0.014	0.50(0.21–1.17)	0.108
2–18	21(25.0)	78(33.3)	1.0		1.0	
Education level						
Secondary	51(60.7)	56(23.9)	5.77(2.2514.78)**	0.001	4.3(1.34–13.91)*	0.014
Primary	27(32.1)	140(59.8)	1.22(0.47–3.17)	0.681	0.87(0.31–2.48)	0.801
None	6(7.1)	38(16.2)	1.0		1.0	
Occupation						
Employed	58(69.1)	116(49.6)	2.27(1.34–3.85)*	0.002		
Not employed	56(66.7)	118(50.4)	1.0			
SES Quintiles						
High	28(33.3)	26(11.1)	4.0(2.17–7.36)**	0.001	2.13(1.03–4.41)*	0.042
Low	56(66.7)	208(88.9)	1.0		1.0	
Disability						
Mobility	73(86.9)	115(49.1)	6.98(2.88–16.9)**	0.001		
Hearing	5(5.9)	53(22.7)	1.0(0.30–3.59)	0.953		
Vision speech	6(7.1)	66(28.2)	1.0			
Awareness						
Yes	59(70.2)	59(25.5)	7.0(4.0312.17)**	0.001	5.1(2.74–9.54)**	0.001
No	25(29.8)	175(75.8)	1.0		1.0	
Nature						
Acquired	77(91.7)	185(79.1)	2.91(1.26–6.72)*	0.012		
Congenital	7(8.3)	49(20.9)	1.0			
Cause of disability						
Others	11(13.1)	48(20.5)	0.2(0.10–0.45)**	0.001		
Infection	20(23.8)	138(59.0)	0.13(0.710.24)**	0.001		
Road traffic crash	53(63.1)	48(20.5)	1.0			

**Multivariate analysis, 95% Confidence interval, *P* value< 0.05 are statistically significant

After performing multivariate analysis, variables that were significantly associated with utilization of rehabilitation services included; age (AOR: 0.30; 95% CI: 0.12–0.74), socioeconomic status (AOR: 2.13; 95% CI: 1.03–4.41), education level (AOR: 4.3; 95% CI: 1.34–13.91) and awareness of the participants about the services (AOR: 5.1; 95% CI: 2.74–9.54) p value ≤0.05, (Table 4).

After adjusting for socioeconomic status, education level and awareness of the participants about the services, people with physical disabilities aged 34 years and above had 70% less odds of utilizing rehabilitation services than those aged 2–18 years (AOR: 0.30; 95% CI: 0.12–0.74; *p* = 0.009). After adjusting for age, socioeconomic status, and awareness of the participants about the services, the odds of utilizing rehabilitation services was 4.3 times higher among people with disabilities who had attained secondary education compared to those no education (AOR: 4.3; 95% CI: 1.34–13.91; *p* = 0.014). After adjusting for age, education level, and awareness of the participants about the services, the odds of utilizing rehabilitation services was 2.1 times higher among people with disabilities from higher socio economic quintile compared to those from low socio economic quintile (AOR: 2.13; 95% CI: 1.03–4.41; *p* = 0.042). After adjusting for age, education level, and socioeconomic status, people with physical disabilities who were aware of rehabilitation services had 5.1 times more odds of utilizing rehabilitation services than those who were not aware of the services (AOR: 5.1; 95% CI: 2.74–9.54; *p* = 0.001).

## Discussion

This study revealed a prevalence of 26.42% of the utilization rehabilitation services among people with physical disability in the study area. According to WHO’s report, rehabilitation is an essential component of quality health services that should be included in Universal Health Coverage. This means that all individuals should be able to access quality rehabilitation services without fear of financial hardships [3]. Therefore, the prevalence of utilization of rehabilitation services was considered very low in the study area. The prevalence of utilization of rehabilitation services obtained in this study is almost consistent with other studies conducted in Southern Brazil [36], South Africa [37], and Beijing China [38] with the prevalence of 20, 22.5 and 27% respectively. The result of this study was much lower than the previous studies conducted among people with physical disabilities in Lages state of Santa Catarina [35] and Geneva Switzerland [39] with the prevalence of 33.2 and 37.2% respectively, but higher than previous studies conducted in Brazil [40], USA [41] and Taiwan [42] with the prevalence of 14.5,6.8 and 11.02% respectively. Socioeconomic and geographical variations might be the reason for the different prevalence’s of the utilization of rehabilitation services among people with physical disabilities across countries and regions.

In the present study, majority (69.5%) of the study respondents were male. The finding is consistent with a previous study conducted in South Africa [37] but inconsistent with a population based survey conducted in Uganda which showed that disability was higher among women compared to men [29]. This could be because the present study was conducted in an urban area where men got involved in many occupational activities like motorcyclists, taxi drivers and street vending which increases their risk of getting involved in accidents thus sustaining injuries and disabilities. In addition, Uganda has been reported to have high rate of accidents among commercial motorcyclists with Mulago hospital alone receiving five (5) to twenty (20) accident victims of commercial motorcyclists on a daily basis [43].

In this study, age, socioeconomic status, education level and awareness of the participants about the services were significantly associated with utilization of physical rehabilitation services.

In this study, age was significantly associated with utilization of rehabilitation services and people with physical disability aged 34 years and above had 70% less odds of utilizing physical rehabilitation services than those aged 2–18 years. This could be because elders are unlikely to utilize the physical rehabilitation services due to poor socioeconomic status, low education level, high rate of unemployment and long distance to the facility which is evident in this study. The study findings are consistent with previous studies conducted in United States of America [41], South Africa [37] which reported low level of utilization of rehabilitation services among elderly population but inconsistent with other studies conducted in Brazil [35, 36] and China [42] which revealed that the use of rehabilitation services was higher among elders aged 35–60 years. The difference in study findings could be partly attributed to the variation in the social demographic profile of different countries. Therefore, the government and other relevant stakeholders should deliver community based rehabilitation services to increase access to these services in the communities.

In this study, higher socioeconomic status was significantly associated with increased utilization of rehabilitation services. This could be because participants from higher socioeconomic quintile might have better education and financial ability to utilize rehabilitation services unlike their counterparts from low socioeconomic quintile. Similar results were reported from other studies conducted in India [44] and Brazil [35, 36]. Therefore, the government should establish community based rehabilitation (CBR) programs within the community and provide rehabilitation services in primary health care facilities to reduce the transport costs and increase accessibility to the services.

In the present study, higher education level was significantly associated with increased utilization of rehabilitation services. This could be because participants who had attained higher level of formal education are more likely to have better knowledge about the benefits of utilizing rehabilitation services. This result is consistent with other studies conducted in India [45], China [46] and Brazil [35].Therefore, the government should provide more education opportunities to people with physical disabilities like vocational training and state scholarships targeting people with physical disabilities.

Furthermore, this study revealed that awareness of the participants about rehabilitation services was significantly associated with utilization of rehabilitation services and people with physical disabilities who were aware of physical rehabilitation services had 5.1 times more odds of utilizing physical rehabilitation services than those who were not aware of the service (AOR: 5.1; 95% CI: 2.74–9.54; *p* = 0.001). This could be because participants who were aware of rehabilitation services were more likely to know the benefits, available services and facilities providing such services which could make them to utilize the services unlike their counterparts who were not aware of the services. Similar findings were reported by previous studies conducted in South Africa [37], India [44] and China [47]. Therefore, the government and other non-government organizations should increase sensitization and awareness of rehabilitation services, their benefits, available services and facilities providing such services among people with physical disabilities and the general public.

A number of challenges were also pointed out in the findings of this study. First, it appears that rehabilitation services such as therapeutic exercise modalities, assistive device technologies, hearing aids, speech therapies and vision aids are essentially unknown to people with physical disabilities and health professionals from other disciplines. The study findings are consistent with other studies conducted in Switzerland [48] and Urban Uganda [23]. Given the increasing number of people with disabilities in Uganda, it appears that the task of sensitizing healthcare professionals, policy makers and the public about the available rehabilitative services is of paramount importance. The results of this study might also assist in the modification of programs that have previously been developed and aimed at training individuals to deliver community rehabilitation services.

## Conclusion

The study revealed a prevalence of 26.4% of the utilization rehabilitation services among people with physical disabilities in Kawempe division, Uganda. Factors that were significantly associated with utilization of rehabilitation services included age, socioeconomic status, education level and awareness of the participants about the services. Therefore, the government and other relevant stake holders should increase sensitization and awareness of rehabilitation services to people with physical disabilities, healthcare professionals and the general public, provide more education opportunities to people with physical disabilities, establish a public funding mechanism targeting people with physical disabilities, integrate basic rehabilitation services within the existing health care service delivery and establish community based rehabilitation centers to increase access to rehabilitation services.

## Data Availability

The datasets used or analyzed during the current study are available from the corresponding author on reasonable request.

## Abbreviations

**AOR**: Adjusted Odds Ratio
**CBR**: Community Based Rehabilitation
**COR**: Crude Odds Ratio
**CRPD**: Convention of the Rights of Persons with Disabilities
**DALYs**: Disability Adjusted Life Years
**KCCA**: Kampala Capital City Authority
**LMICs**: Low and Middle-Income Countries
**MOH**: Ministry of Health
**NUDIPU**: National Union of Disabled Persons of Uganda
**PWDs**: People with Disabilities
**PWPDs**: People with Physical Disabilities
**UN**: United Nation
**UNAPD**: Uganda National Action on Physical Disability
**WHO**: World Health Organization
